# Investigation into scalable and efficient enterotoxigenic *Escherichia coli* bacteriophage production

**DOI:** 10.1038/s41598-024-53276-w

**Published:** 2024-02-13

**Authors:** Katie G. Wiebe, Bradley W. M. Cook, Tasia J. Lightly, Deborah A. Court, Steven S. Theriault

**Affiliations:** 1 Cytophage Technologies Inc., Winnipeg, MB Canada; 2 Department of Microbiology, University of Manitoba, Winnipeg, MB Canada

**Keywords:** Bacteriophages, Industrial microbiology, Virology

## Abstract

As the demand for bacteriophage (phage) therapy increases due to antibiotic resistance in microbial pathogens, strategies and methods for increased efficiency, large-scale phage production need to be determined. To date, very little has been published on how to establish scalable production for phages, while achieving and maintaining a high titer in an economical manner. The present work outlines a phage production strategy using an enterotoxigenic *Escherichia coli*-targeting phage, ‘Phage75’, and accounts for the following variables: infection load, multiplicity of infection, temperature, media composition, harvest time, and host bacteria. To streamline this process, variables impacting phage propagation were screened through a high-throughput assay monitoring optical density at 600 nm (OD_600_) to indirectly infer phage production from host cell lysis. Following screening, propagation conditions were translated in a scalable fashion in shake flasks at 0.01 L, 0.1 L, and 1 L. A final, proof-of-concept production was then carried out in a CellMaker bioreactor to represent practical application at an industrial level. Phage titers were obtained in the range of 9.5–10.1 log_10_ PFU/mL with no significant difference between yields from shake flasks and CellMaker. Overall, this suggests that the methodology for scalable processing is reliable for translating into large-scale phage production.

## Introduction

Bacteriophages (phages) are viruses that target and infect bacteria. Lytic or virulent phages are considered alternative therapeutics to antibiotics for the prevention and treatment of bacterial disease^1^. Due to the prevalence of antibiotic resistance, studies have been carried out using both standalone phage therapy and combinations of phages and antibiotics to treat bacterial infections in humans and other animals^1–8^. However, for phage therapy to become more realistic on a global scale, efficient strategies to mass produce phages need to be established^9,10^.

High-throughput methods in phage research have been used to assess phage cocktail efficiency^11^, phage virulence^12^, phage host range^13^, and optimal multiplicity of infection (MOI) conditions^14^, demonstrating their value in streamlining experiments. Additionally, screening assays have been used as a proxy to quantify phages over time by monitoring optical density at 600 nm (OD_600_) wavelength as it inversely relates to host lysis; a decrease in OD_600_ is an indication of cell lysis and therefore phage production^15^. High-throughput assays can offer invaluable insight into the initial stages of a phage production strategy, allowing for predictions regarding optimal conditions for achieving a high phage titer. This approach will save time and costs by eliminating unfavourable conditions early in the process.

Often phage production strategies start out at small scale with shake flasks to identify optimal conditions for achieving maximal phage titer before being scaled-up to a bioreactor system^16^. However, phage production cannot always be effectively scaled-up from the laboratory scale, possibly due to infeasibility of the method, inability to keep conditions and optimized parameters constant, and/or the diverse nature of phages and phage-host dynamics during production at larger volumes^17–19^. To help ensure a scalable process without loss of titer, bioreactor-specific conditions may be optimized early on, during process development^18,20^, with typical volume increments by factors of 10 or less^17^.

Considering infection conditions, infection load (the amount of bacterial culture added in percent volume/volume) and MOI (ratio of infectious phages to viable bacterial cells) influence phage-host dynamics and the number of infection cycles that take place over the course of production^9,20,21^. An intermediate MOI may delay lysis, thereby allowing bacterial hosts to continue producing phages; in contrast an overly high or low MOI, results in rapid lysis or unimpeded bacterial growth respectively, sacrificing phage titer^14,15,21^. Growth conditions such as media composition^9,14,22^, supplementation of ions^14,23^, and temperature^16,24^ can impact phage production^20^. Media formulations require careful consideration in large-scale propagation as defined media may alleviate issues relating to high costs, animal-sourced components, batch-to-batch variability, and more complex downstream processing associated with complex media^9,25–27^.

Different harvest times, ranging from hours to overnight, have been described in the literature, although the reason for this is not always specified^28–33^. Ideally, phage would be harvested when population-wide lysis of the bacterial host is observed, typically by decreased optical density, indicating peak phage titer has been achieved^20^. Extending production past peak titer may result in little to no improvement and potentially result in a decreased titer^22,34^. Therefore, monitoring titer over time is important in establishing efficient, time-saving phage production.

With respect to phage therapy, although the targets are clinical isolates, a less pathogenic host can be considered for phage propagation, thus limiting undesirable traits such as virulence, toxin production, and multi-drug resistance^19,35^. Surrogate hosts that follow guidelines summarized by Merabishvili et al*.,* 2018 are recommended for phage production^35^, leading to safer and simpler processing^36^.

Large scale phage propagations are commonly performed in bioreactor systems involving bacterial growth followed by phage infection within the same vessel^20^. This type of batch operation is considered inexpensive, simple, and robust, with short runs capable of yielding titers between 10^10^ to 10^16^ PFU/mL^3,21,37^. However, the long downtime periods in-between production runs that are necessary for proper cleaning and sterilization are significant disadvantages^3^. Single-use or disposable bioreactors may provide a solution and, unlike traditional bioreactors, use a gentler mixing strategy: aeration to reduce shear stress during the infection process instead of impellers^38^.

In this study, the utility of defining production conditions prior to the transition to large scale phage propagation was investigated using a clinical ETEC disease-causing isolate and newly isolated phage. Information gained from high-throughput screening assays and increasing volumes in shake flasks enabled refinement of the culture conditions in preparation for production in a disposable bioreactor as a representation of industrial scale phage manufacturing.

## Methods

### Bacteria, phages, and culture media

Bacteria and Phages: Enterotoxigenic *Escherichia coli* strain 54 (ETEC54), virotype F41:LT:STa:STb, was isolated from the small intestine of a pig and is the target host of Phage75. Phage75 has a 169 kb dsDNA genome and belongs to the *Tequatrovirus* genus. Phage-host combination, Tequatrovirus T4 (ATCC 11303-B4), herein referred to as T4, and *Escherichia coli* B (ATCC 11303) were used in harvest time experiments to compare to Phage75 and ETEC54. *Escherichia coli* C (ATCC 13706) was explored as a surrogate host for Phage75 production. All bacterial strains were grown aerobically with agitation at 37 °C. ETEC54 is considered a containment level 2 strain while *E. coli* B and C are containment level 1 strains.

Culture Media: Several different media were investigated and used for bacterial growth and phage production: LB-Lennox (LB) (10 g/L tryptone, 5 g/L yeast extract, 5 g/L sodium chloride), Select APS (APS) (2.5 g/L soy hydrolysate, 12.5 g/L yeast extract, 5 g/L sodium chloride), SM-1 novel (SM-1)[10 g/L casamino acids, 3.5 g K_2_HPO_4_, 5 g Na_2_SO_4_, 1% glucose, pH = 7.2 ± 0.2 (modified from^27^)], M9 minimal (M9) [200 mL of M9 minimal salts 5x, 2 mL 1 M MgSO_4_, 100 µL 1 M CaCl_2_, 0.4% (w/v) glucose, per 1 L^39^], and Optimized M9 minimal (Opt. M9) [200 mL of M9 minimal salts 5×, 2 mL 1 M MgSO_4_, 100 µL 1 M CaCl_2_, 0.8% (w/v) glucose, 0.4% (w/v) tryptone, per 1 L (modified from^40^)].

### Phage titration

The double-agar overlay (plaque assay) technique adapted from Kropinski et al*.,* 2009 was used to titrate phage production samples. Briefly, 3 mL of molten, soft agar comprised of LB containing either 0.2% agarose (Phage75) or 0.6% agar (T4), 200 µL of respective host bacteria and 100 µL of phage sample dilution were mixed and then pour-plated onto 100 mm Petri dishes containing a solidified LB 1.5% agar underlay. Plaques were counted following overnight incubation at 37 °C. For harvest time experiments, an adapted drop-plating protocol was employed^41^. Briefly, 3 mL of molten, soft agar overlays containing LB with either 0.2% agarose (Phage75) or 0.6% agar (T4) and 200 µL of host bacteria were mixed and pour-plated onto Petri dishes and allowed to solidify for approximately 15 min. Then, 10 µL drops of diluted phages samples were spotted onto the soft agar overlay and allowed to dry prior to incubation. Plaques were counted following overnight incubation at 37°C. Phages were quantified as plaque-forming unit (PFU)/mL and transformed to log_10_ PFU/mL for graphing and subsequent analysis.

### Efficiency of plating (EOP)

Using the double-agar overlay protocol described above, Phage75 was quantified on different bacterial hosts: ETEC54, *E. coli* C, and *E. coli* B. The EOP was calculated by dividing average titer obtained either on *E. coli* C or *E. coli* B by the average titer on ETEC54 and then multiplying by 100^42^.

### High-throughput screening plate assay

A 96-well plate assay based on Rajnovic et al*.,* 2019, was designed to screen production variables for Phage75 production. Bacterial cultures were started from a single colony and incubated until an OD_600_ of 0.6 was reached in each media type, and then diluted 1/10 which corresponds to 10^7^ colony-forming unit (CFU)/mL. Phage stocks were diluted in the same media to appropriate MOI(s) to be tested. To 160 µL of media, 20 µL of bacterial host and 20 µL phage dilutions were added to each well (200 µL per well final volume). To prevent dehydration, the perimeter wells contained sterile water. Plates were incubated overnight in a plate reader (Biotek EPOCH 2 or Biotek Synergy HTX) set to continuous orbital shaking 205 cycles per minute (cpm) (5 mm) with OD_600_ measurements taken in 15-min intervals. Assays for infection (exception: infection load) and operation conditions were repeated with a series of MOI (10^–6^ to 1), media types (LB, APS, SM-1, M9 minimal, and Opt. M9), temperatures (28 °C, 33 °C, 37 °C, and 40 °C), and ion supplementations (0–10 mM of CaCl_2_ or MgSO_4_ in LB).

### Shake flask productions

All cultures regardless of volume were prepared by inoculating 1–3 bacterial colonies into fresh media and then incubated in a shaking incubator at 37 °C until an OD_600_ 0.6, corresponding to 10^8^ CFU/mL and 10^7^ CFU/mL for ETEC54 and *E. coli* B respectively. For *E. coli* C, cultures were incubated until OD_600_ of 0.6 in LB and OD_600_ of 0.8 in APS were reached to ensure a bacterial concentration of approximately 5.5 × 10^7^ CFU/mL. Then, respective phages (Phage75 and T4) were diluted to the appropriate MOI and inoculated into fresh media along with cultures.

Infection conditions: Infection load (IL) was investigated at 0.01 L volumes in 50 mL shake flasks with 1 to 5%, 7%, 10%, and 20% considered for both MOI 10^–3^ and 10^–4^. Shake flasks were agitated at 200 rpm in a mini-orbital shaker (VWR, Mississauga, Canada) for 8 h at 37°C. One mL samples were obtained, filter sterilized in 0.2 µm syringe filters (VWR, Mississauga, Canada, and, UltiDent Scientific, Montreal, Canada) and titrated via double-agar overlay technique to determine phage titer. A 4% IL was chosen and applied in all subsequent scales of production. For 0.1 L and 1 L volumes in 500 mL and 2 L flasks respectively, productions were incubated at 37 °C unless otherwise specified, with 160 rpm and 120 rpm, respective agitation speeds in an Excella E24 orbital shaking incubator (VWR, Mississauga, Canada). Like the 0.01 L productions, 1 mL samples were collected, filter sterilized, and titrated via double-agar plaque assay. For harvest-time experiments, flasks were prepared as described above. Every hour for 8 h and a final overnight sample at 22 h (only at 1 L scale), 1 mL samples were obtained, filter sterilized, and titrated with drop-plating plaque assays. Additional OD_600_ measurements were taken at 30-min intervals (Biochrom WPA CO8000 cell density meter, Montreal Biotech, Dorval, Canada).

### CellMaker bioreactor

A single, proof-of-concept 3 L production of Phage75 was carried out in the CellMaker bioreactor, 8 L enclosure (Cellexus, Scotland). Autoclaved LB broth was pumped into a bioreactor bag through a Versapor pleated 0.2–0.8 µm filter using a peristaltic pump (Masterflex, Germany) and heated to 37°C. Using optimized conditions from shake flask productions, a 4% IL and MOI of 10^–4^ was achieved by adding 120 mL of ETEC54 culture (OD_600_ of 0.6) and 30 mL of Phage75 (5.3 × 10^4^ PFU/mL) to the bioreactor bag by syringe. The airflow of the sparge tube was set to 0.25 L per minute. Aliquots were removed in triplicate for titration from sample ports using syringes at 3- and 4-h timepoints.

### Statistical analysis

The mean titer of Phage75 and T4 from productions were analyzed through GraphPad Prism 10.0.3 (Dotmatics, San Diego) using one-way ANOVA with an alpha threshold of 0.05 and 95% confidence interval and followed with Tukey multiple comparisons test to assess significance of specific production conditions.

## Results

### Identifying production conditions

**Figure 1:**
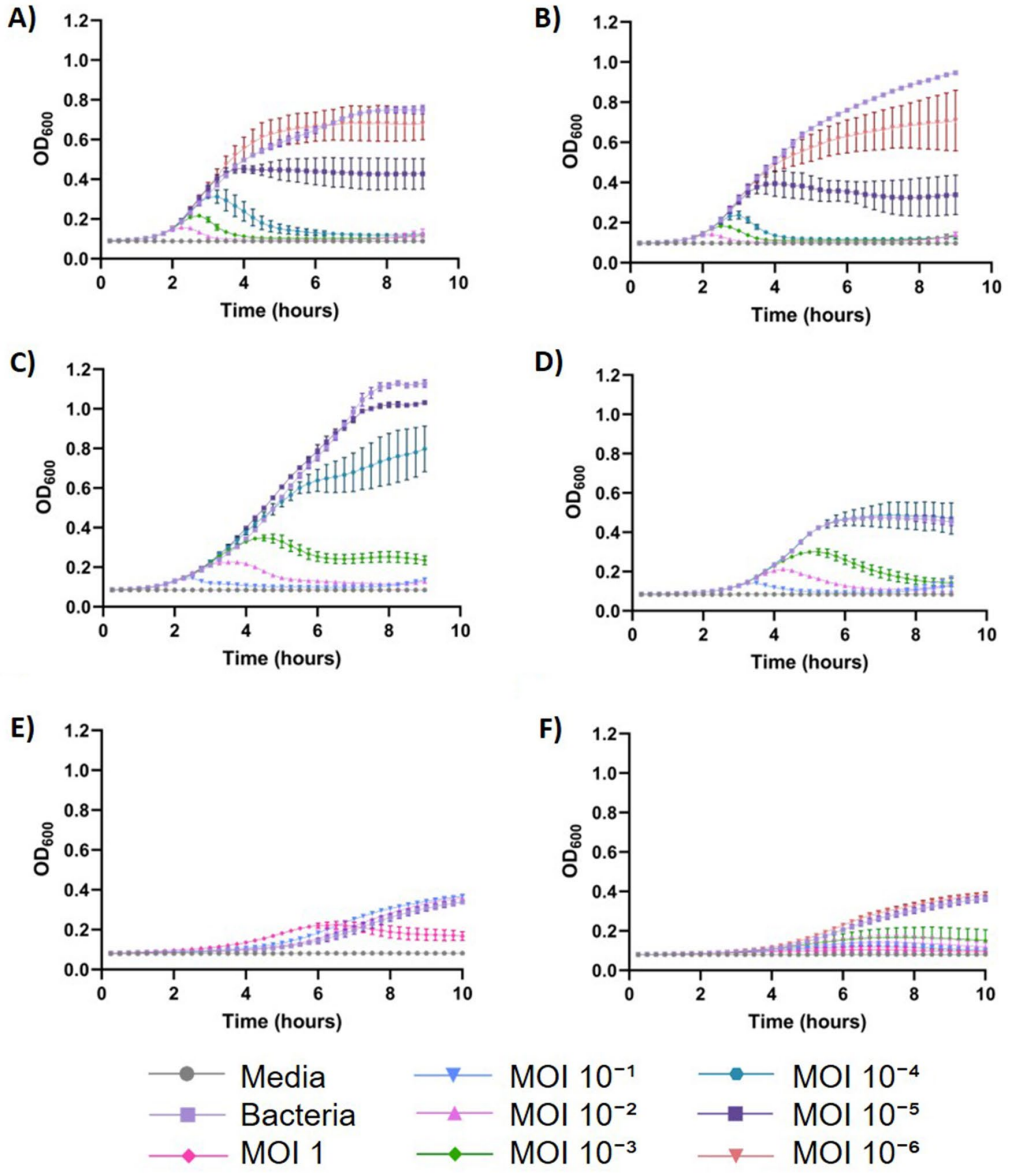
Effect of media on bacterial growth in the presence of phage at different MOI. (**A**–**E**) ETEC54 and Phage75; (**F**) *E. coli B* and T4. Media types: (**A**) LB; (**B**) APS; (**C**) Opt. M9; (**D**) SM-1; (**E**, **F**) M9 minimal. Error bars show standard deviation from the mean (n = 3).

A high-throughput screening assay to analyze bacterial growth reduction as a proxy for phage cultivation was used to define conditions for maximal Phage75 production using ETEC54 as the host. Conditions which offered a balance between bacterial growth and lysis, indicated by increasing and decreasing OD_600_ values, were further pursued in subsequent screening assays to establish optimal conditions for future scale-up (Table 1). For infection conditions, only MOI was assessed in this assay and infection load testing was performed at the 0.01 L scale. Operating conditions (media, temperature, harvest time, and ion supplementation) were also analyzed with this assay. The influence of MOI in the infection conditions suggested that using extremes would likely have a negative impact on phage titer, causing rapid host lysis or unimpeded growth from high or low MOI respectively and was also affected by media choice (Fig. 1). Specifically in LB, MOI 10^–2^ and 10^–3^ were too high and didn’t allow much bacterial growth prior to lysis, and MOI 10^–5^ and 10^–6^ were too low, allowing ETEC54 to continually grow (Fig. 1A). However, a 10^–4^ MOI condition allowed for the highest OD (0.31) to be reached in LB and lysis occurred approximately at 5 h. Similar trends were seen in APS, though maximum OD for the 10^–4^ MOI condition was 0.24 and lysis occurred at around 4 h (Fig. 1B). Because of this reduction, a 10^–5^ MOI condition was additionally considered in APS for comparison with the 10^–4^ MOI condition. Screening MOI in Opt. M9 revealed that MOI conditions 10^–1^ and 10^–2^ led to quick lysis, with 10^–3^ offering a balance between growth and lysis, and MOIs 10^–4^ and 10^–5^ showing high bacterial growth (Fig. 1C). During SM-1 screens, the lowest MOI to result in lysis was 10^–3^, as a MOI of 10^–4^ already resembled that of the bacterial control (Fig. 1D). Interestingly, in M9 minimal medium, only under a MOI of 1 was there a reduction in growth of ETEC54 (Fig. 1E). As a process control, the assay was repeated using a different phage-host pair, T4 and *E. coli* B. This screen revealed that a MOI of 10^–3^ sufficiently depleted *E. coli* B, suggesting M9 minimal supports T4 production better than that of Phage75 (Fig. 1F). For operating conditions, 33°C or 37°C allowed more bacterial growth prior to lysis than at 28°C and 40°C, with an exception in Opt. M9, indicating more host cells were available during propagation and would likely lead to higher phage yields (Supplementary Fig. S1). Additionally, these assays provided insight as to possible harvest times for production based on ETEC54 lysis and corresponding drop in OD_600_ which resembled that of the media control (Supplementary Fig. S1). Divalent cation sources, CaCl_2_ or MgSO_4_, were also screened in assays to determine if their supplementation could potentially improve Phage75 production, however results did not reveal a clear benefit (Supplementary Fig. S2) and therefore, ion supplementation was excluded from further analysis. Thus, LB (MOI 10^–4^), APS (MOI 10^–4^ and 10^–5^), SM-1 (10^–3^), M9 (MOI 1), and Opt. M9 (10^–3^) were selected for small scale propagation.

**Table 1:** Summary of predicted Phage75 production conditions based on screening assays.

	LB-Lennox (LB)	Select APS (APS)	SM-1 novel	M9 minimal	Optimized M9 (Opt. M9)
MOI	10^–4^	10^–4^ & 10^–5^	10^–3^	1	10^–3^
Temperature	33 °C or 37 °C	37 °C	37 °C	Not tested	33 °C or 37 °C
Harvest time	4.5–5 h	4.5–5 h	8 h	Undetermined	6 h

No obvious benefit to divalent cation (CaCl_2_ or MgSO_4_) supplementation.

### Small-scale productions

Translation from small to medium to large scale (as defined in this study) was assessed with optimal MOI, media, and temperature conditions in 0.01 L, 0.1 L, and 1 L culture volumes. However, some additional investigations were necessary to narrow down the best conditions for maximum phage production. As part of the infection control parameters, infection load (IL) (volume of host cell culture used to start phage production) is important as it relates to the number of bacterial cells initially present, and may affect the number of phage replication cycles and influence phage yields^20^. The decision to simplify this was made using LB medium and two MOIs (10^–3^ and 10^–4^) to address any influence that load volumes had on overall titers at 0.01 L. Despite IL ranges from 1 to 20%, the average Phage75 titers produced were similar; except for 20% which led to a reduction of approximately 1.5 log_10_ PFU/mL, which was statistically significant compared to all other conditions tested (*p* $\le$ 0.0004, Supplementary Fig. S3). Therefore, a decision to reduce MOI to 10^–4^ and use an intermediate IL of 4% was chosen for further scale-up testing.

**Figure 2:**
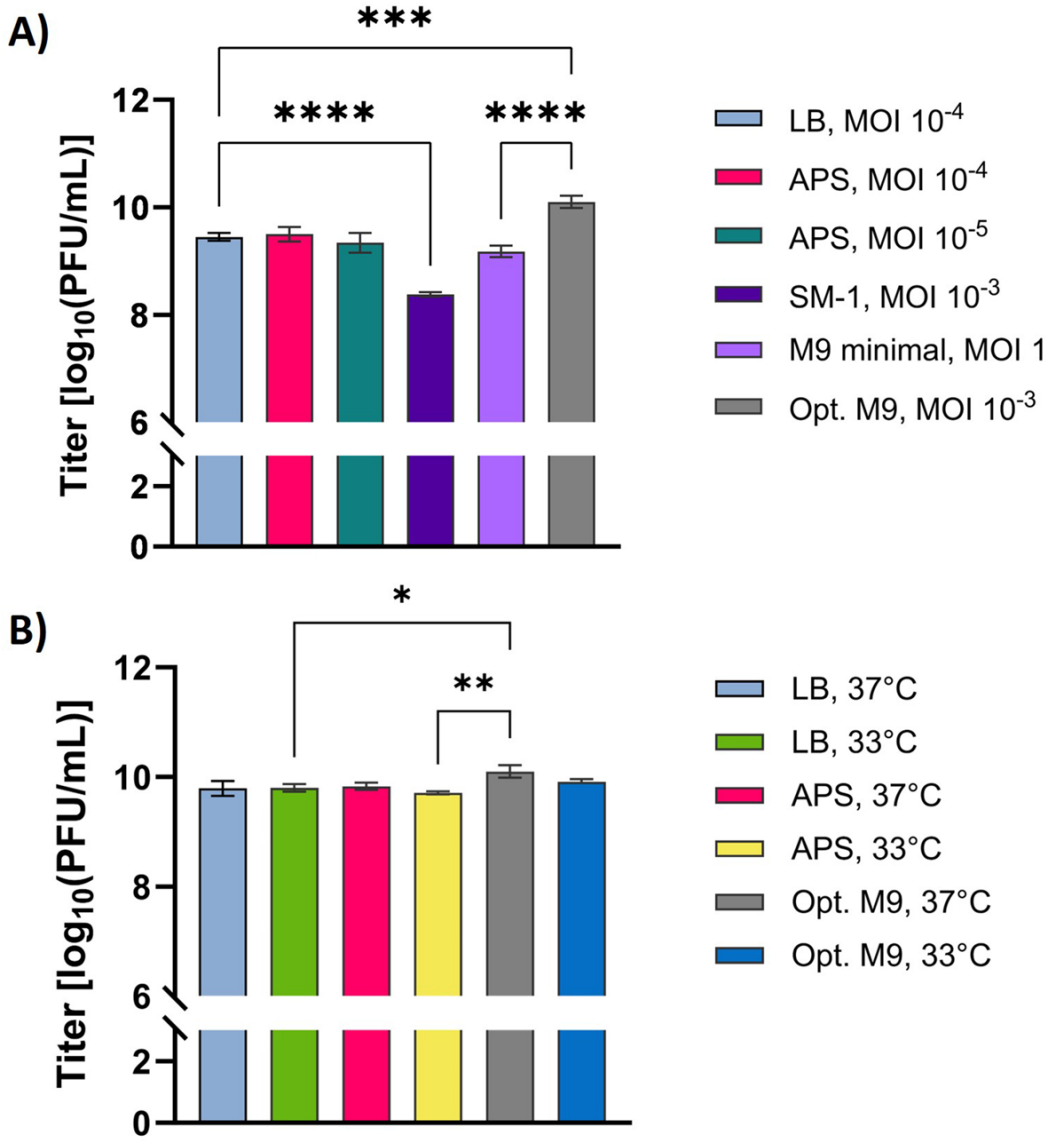
Titers for small-scale productions of Phage75 with ETEC54. (**A**) Comparison of “best” MOI condition(s) for each medium. (**B**) Comparison of 37°C and 33°C for most promising medium types: LB; APS; Opt. M9. Media (MOI): LB (10^–4^); APS (10^–4^); Opt. M9 (10^–3^). Note: average titer of Opt. M9, 37°C production displayed in (**A**) was duplicated and included in (**B**). Error bars show standard deviation from the mean (n = 3). Statistics performed using one-way ANOVA with Tukey multiple comparisons test, ****p $<$ 0.0001; *** p = 0.0002; ** p = 0.0013; * p = 0.0101.

Moving forward, conditions predicted to yield high titers of Phage75 from plate screening assays were translated to shake flasks with two independent 0.01 L productions carried out for 8 h. The first set of productions compared optimal MOI in different media at 37°C and the second set considered a temperature reduction from 37 to 33 °C, based on OD_600_ reductions from the previous experiments. The highest titers were obtained from Opt. M9 (10.10 ± 0.12 log_10_ PFU/mL) whereas they were similar in LB at MOI 10^–4^ (9.45 ± 0.07 log_10_ PFU/mL), APS at MOI 10^–4^ (9.50 ± 0.14 log_10_ PFU/mL), APS at MOI 10^–5^ (9.34 ± 0.18 log_10_ PFU/mL), and M9 at MOI 1 (9.18 ± 0.11 log_10_ PFU/mL), but was the lowest in SM-1 at MOI 10^–3^ (8.38 ± 0.04 log_10_ PFU/mL) (Fig. 2A). Thus, LB (MOI 10^–4^), APS (MOI 10^–4^) and Opt. M9 (MOI 10^–3^) were compared with respect to temperature changes. A temperature change from 37 to 33°C did not have a significant effect on phage titer in any medium: LB (*p* > 0.9999), APS (*p* = 0.5433), or Opt. M9 (comparing with data point from Fig. 2A; *p* = 0.1400) (Fig. 2B). Significance was seen between Opt. M9 at 37 °C with LB at 33 °C (*p* = 0.0101) and APS at 33 °C (*p* = 0.0013), as well as between LB 37 °C (*p* = 0.0080) and APS 37 °C (*p* = 0.0209).

#### Medium to Large Scale

**Figure 3:**
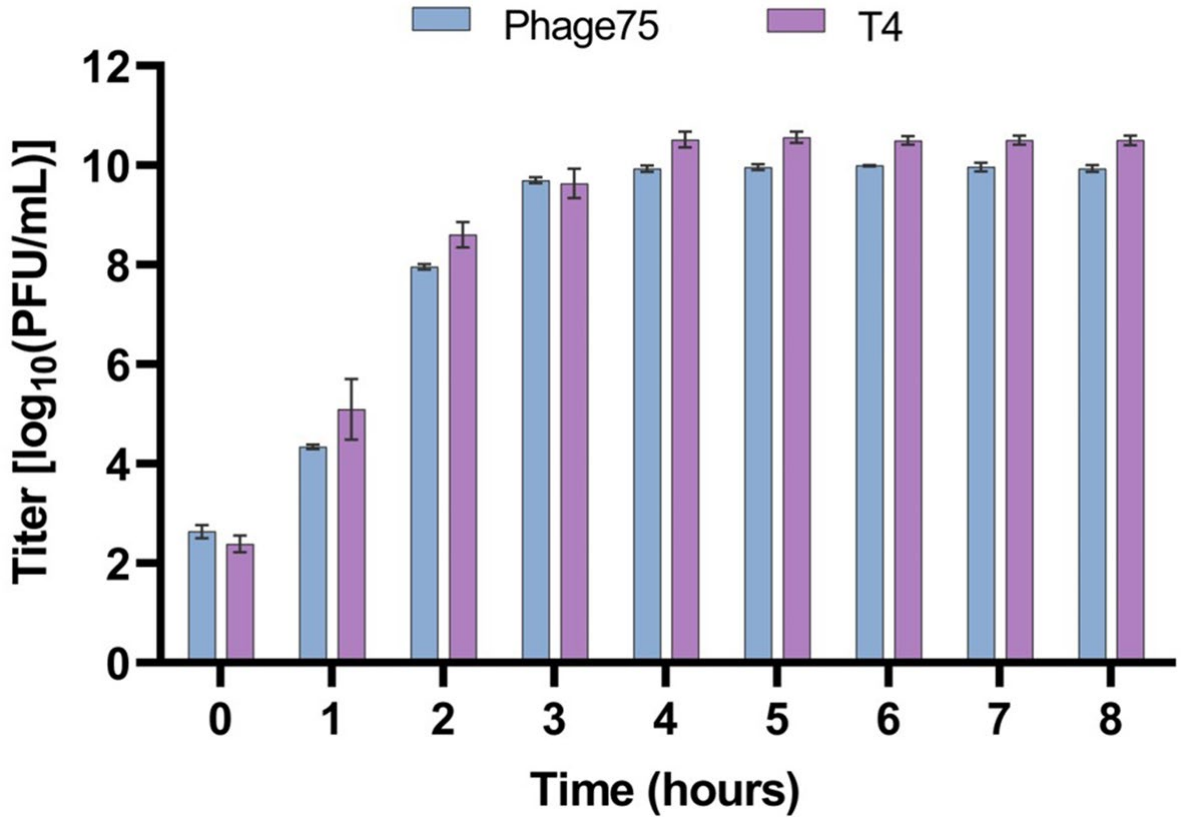
Comparison of phage titer throughout production at 0.1 L scale in LB medium. A 1 mL sample was removed from shake flasks every hour and filter sterilized for titer determination. Error bars show standard deviation from the mean (n = 3).

**Figure 4:**
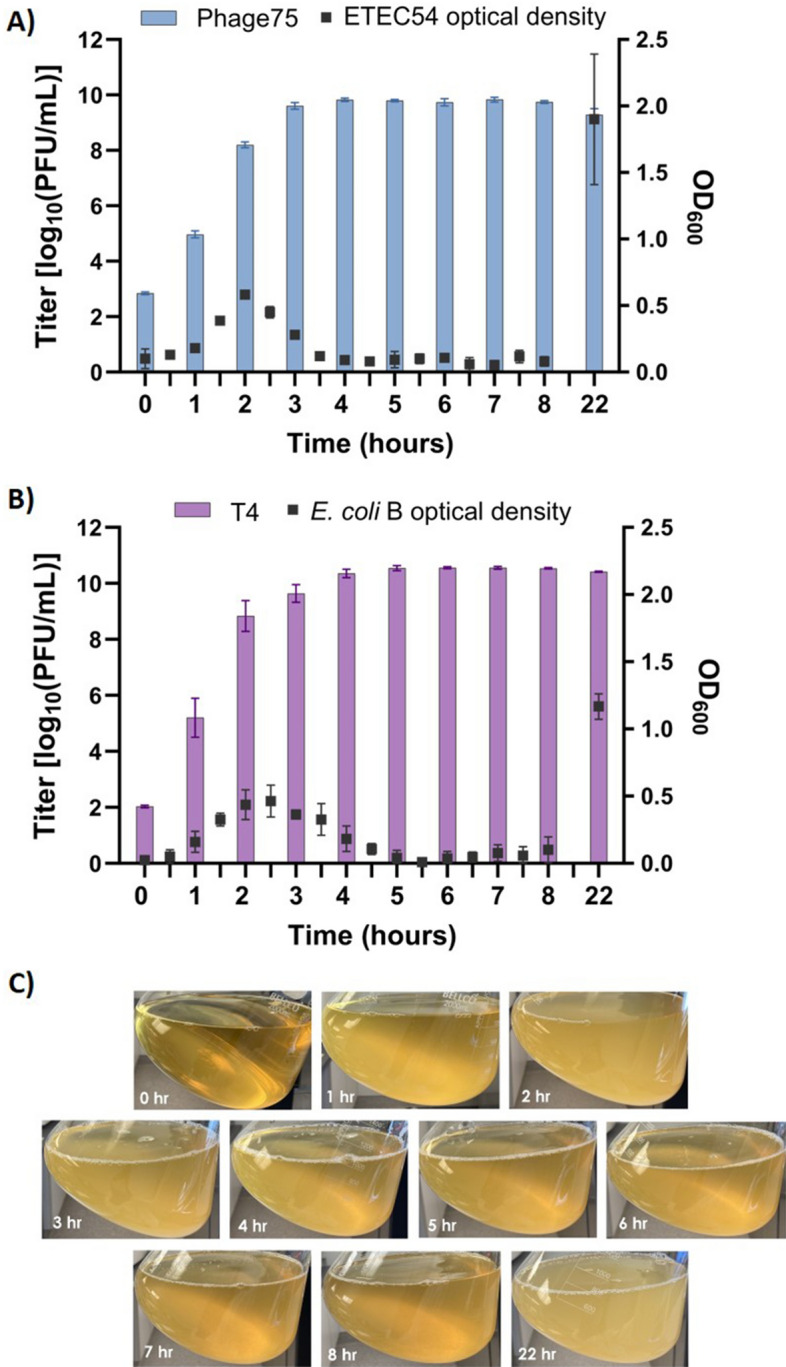
Comparison of phage titer and bacterial optical density (OD) throughout production at 1 L in LB medium. Phage and host combinations at 10^–4^ MOI: (**A**) Phage75 and ETEC54; (**B**) T4 and *E. coli* B. A 1 mL sample was removed from production every hour and filter sterilized for titer determination. Additionally, an OD_600_ measurement was taken every 30 min. Error bars show standard deviation from the mean (n = 3). (**C**) Appearance of 1 L Phage75 production in LB medium over time.

**Figure 5:**
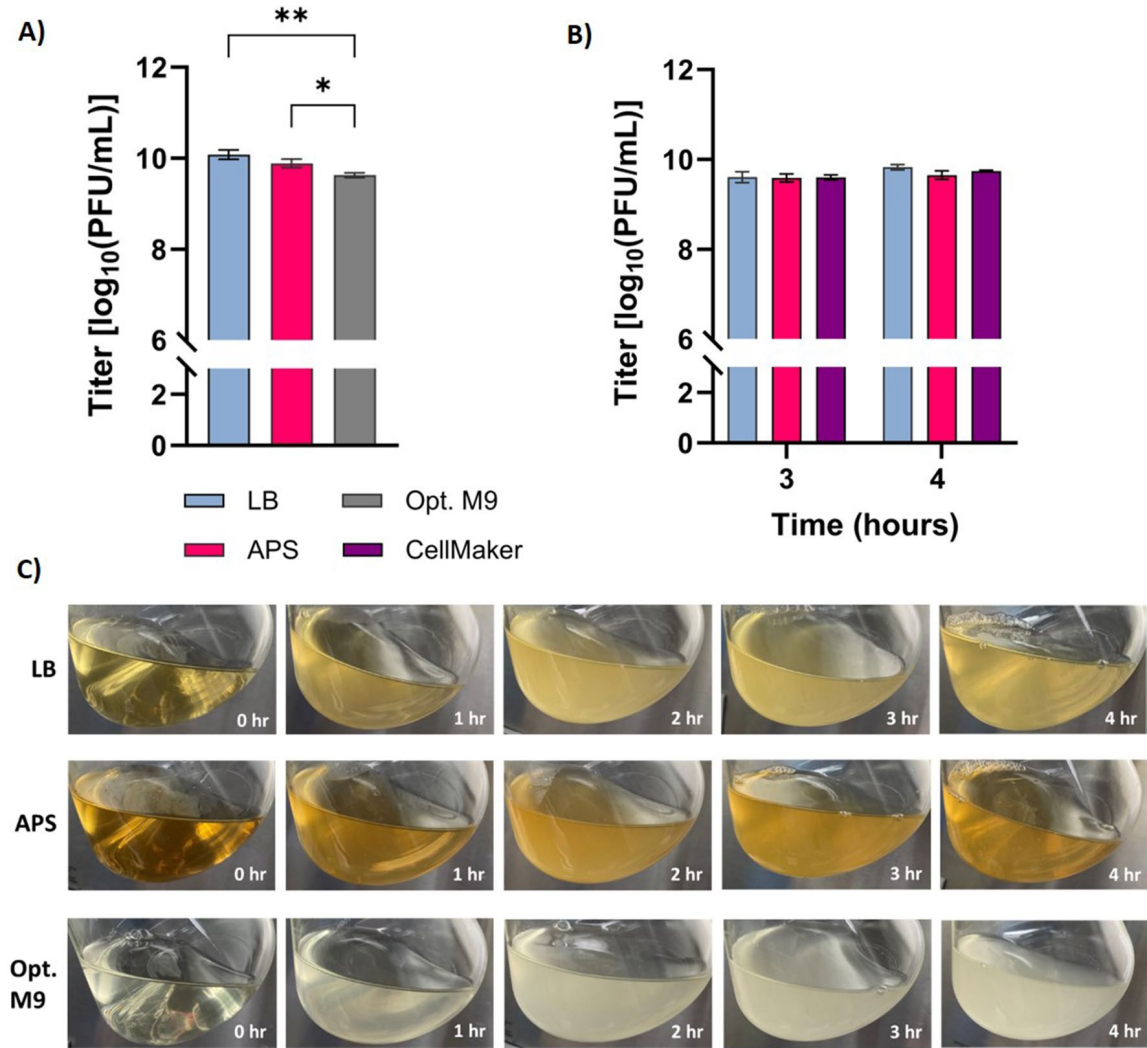
Scaled-up Phage75 productions. All productions were carried out using a 10^–4^ MOI. (**A**) Comparison of media at 0.1 L and harvested at 4 h. (**B**) Comparison between 1 L productions and CellMaker production harvested at 3 or 4 h. Error bars show standard deviation from the mean (n = 3), however for the CellMaker run, n = 1 with error bars rather showing standard deviation among technical replicates. Statistics performed using one-way ANOVA with Tukey multiple comparisons test, ** p = 0.0016; * p = 0.0237. (**C**) Appearance of 0.1 L scale production in different media.

Building onward from the translational aspects of phage propagation, another critical variable is incubation time and when to harvest production. To investigate this, 0.1 L shake flask productions were performed in LB, with a MOI of 10^–4^ at 37°C for 8 h. In addition to Phage75 (ETEC54 host), T4 phage with its *E. coli* B host was used as a control to confirm that the ability to scale up cultures and observed trends are not unique to the phage/host combination. To measure phage production over time, 0.001 aliquots from 0.1 L cultures were collected hourly and titrated via drop plating. The peak titers for Phage75 (9.99 ± 0.01 log_10_ PFU/mL) and T4 (10.56 ± 0.11 log_10_ PFU/mL) were observed at 6 and 5 h-sampling time points respectively (Fig. 3). However, no significant changes in titer were detected from 4 to 8 h for either Phage75 (*p* > 0.97) or T4 (*p* > 0.9999) (Fig. 3). Therefore, this indicated that there was no need to extend production beyond 4 h and delay harvest. The experiment was repeated at 1 L to assess scale-up with phage titrations; OD_600_ measurements and photos were additionally taken to visually verify bacterial lysis. As seen in Fig. 4A, peak phage titers were achieved at 4 h for Phage75 (9.83 ± 0.06 log_10_ PFU/mL) and at 6 h for T4 (10.56 ± 0.03 log_10_ PFU/mL) (Fig. 4B). No significant difference in Phage75 titer was observed in samples taken over 3–8 h of production (*p* = 0.3068 to > 0.9999), again showing a shorter harvest is possible without sacrificing phage titer. A similar trend was observed for T4 production with no significant difference in titer among samples taken over 4 to 8 h (*p* = 0.9982 to > 0.9999). Reaching peak phage titer coincided with the reduction in OD_600_ starting from 0.58 to 0.09 and 0.46 to 0.04 for ETEC54 and *E. coli* B respectively, indicating this can be a suitable cue to harvest phage productions (Fig. 4A,B). Maximum turbidity was detected by OD and visualized at 2 h (Fig. 4A,C), with the largest visual drop in turbidity (Fig. 4C) coinciding with the largest reduction in OD and reaching peak phage titer (Fig. 4A). To address the possibility that results were specific for LB, 0.1 L productions for Phage75 were repeated to include APS and Opt. M9 media while maintaining a 4-h harvest time at 37 °C. Titrations revealed that LB and APS were comparable, 10.10 ± 0.10 and 9.89 ± 0.09 log_10_ PFU/mL (*p* = 0.0765), and Opt. M9 gave the lowest titer 9.62 ± 0.05 log_10_ PFU/mL (*p* = 0.0016 and 0.0237 with LB and APS respectively) (Fig. 5A). Peak phage titer appeared to coincide with the visual decline in turbidity from a maximum observed at 2 h to decline at 4 h post-infection, except for Opt. M9 (Fig. 5C). Thus, due to the reduced titer at the 4-h harvest time, production in Opt. M9 was no longer considered at higher scales. However, due to its similarity in supporting Phage75 production, APS was further scaled-up to 1 L to compare with previous LB productions (harvest time experiment, Fig. 4). Samples were titrated after 3- and 4-h post-infection, corresponding to 9.58 ± 0.09 and 9.65 ± 0.09 log_10_ PFU/mL respectively, whereas in LB, titers at these timepoints had been 9.60 ± 0.12 and 9.83 ± log_10_ PFU/mL respectively (Fig. 5B).

### Bioreactor production

Based on the 1 L production results, scale-up to 3 L in the CellMaker bioreactor was performed in LB to assess whether the proposed method is translatable. Three independent samples were each collected and titrated after 3- and 4-h post-infection, revealing titers of 9.60 ± 0.06 and 9.74 ± 0.02 log_10_ PFU/mL respectively (Fig. 5B). This was consistent with visual interpretation that population-wide lysis occurred between 3.5 to 4 h. Analysis with one-way ANOVA showed no significant difference between titers obtained in shake flasks nor between the CellMaker production and shake flasks (*p* = 0.1004) (Fig. 5B).

### Scalability

**Figure 6:**
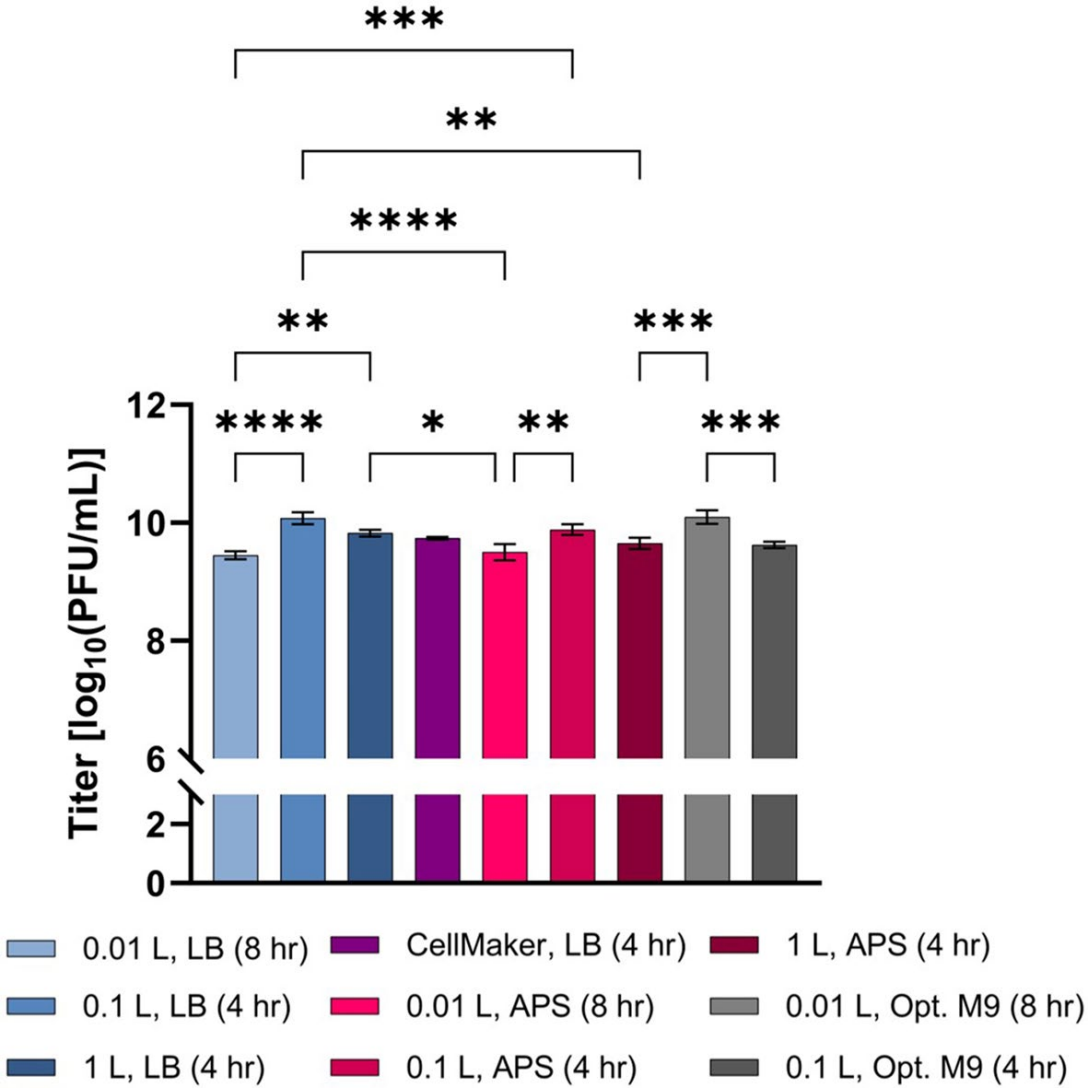
Phage75 production scalability summary. Media (MOI): LB (10^–4^); APS (10^–4^); Opt. M9 (10^–3^). Error bars show standard deviation from the mean (n = 3), however for the CellMaker run, n = 1 with error bars rather showing standard deviation among technical replicates. Statistics performed using one-way ANOVA with Tukey multiple comparisons test, ****p $<$ 0.0001; ***p $\le$ 0.001; **p $<$ 0.005. Only comparisons between different scales shown.

To determine the success of scale-up, productions in LB, APS, and Opt. M9 were compared from 0.01 to 1 L, regardless of harvest time (Fig. 6). Among productions carried out in LB, titers obtained at the 0.01 L scale were significantly lower than those obtained either at the 0.1 L or 1 L scale (*p* =  < 0.0001 and 0.0042 respectively), however no significance was seen between the 0.1 L and 1 L scales (*p* = 0.0855). In APS, the average titer achieved during production at 0.01 L was significantly lower than that achieved at 0.1 L (*p* = 0.0033), though no significant difference was seen between 0.01 and 1 L, nor between 0.1 and 1 L (*p* = 0.5964 and *p* = 0.1287 respectively). Production in Opt. M9 resulted in a significant titer reduction at the 0.1 L scale compared to the 0.01 L scale (*p* = 0.0004). Comparing production titer in Opt. M9 at the 0.01 L scale to 0.1 L scale in either LB or APS, and to 1 L LB showed no significance (*p* > 0.05), though a significant difference was seen when titer was compared to production in 1 L APS (*p* = 0.0007). However, differences in titer between productions in Opt. M9 at 0.1 L compared to those carried out in either LB or APS at 0.01 L or 1 L were not significant (*p* = 0.2586 to > 0.9999). Significance was seen between productions in APS at 0.01 L to productions in LB at 0.1 L and 1 L (*p* =  < 0.0001 and 0.0146 respectively), as well as between productions in 0.01 L LB and 0.1 L APS (*p* = 0.0010) and between 0.1 L LB to 1 L APS (*p* = 0.0012). There was no significant difference in titer between productions 0.1 L APS and 1 L LB nor between 1 L APS and 0.01 L LB (*p* = 0.9962 and *p* = 0.2624). Additionally, no significant difference in titer was observed between the CellMaker production and all other productions included in this comparison analysis (*p* = 0.0778 to 0.9955) (Fig. 6).

### Surrogate host

**Figure 7:**
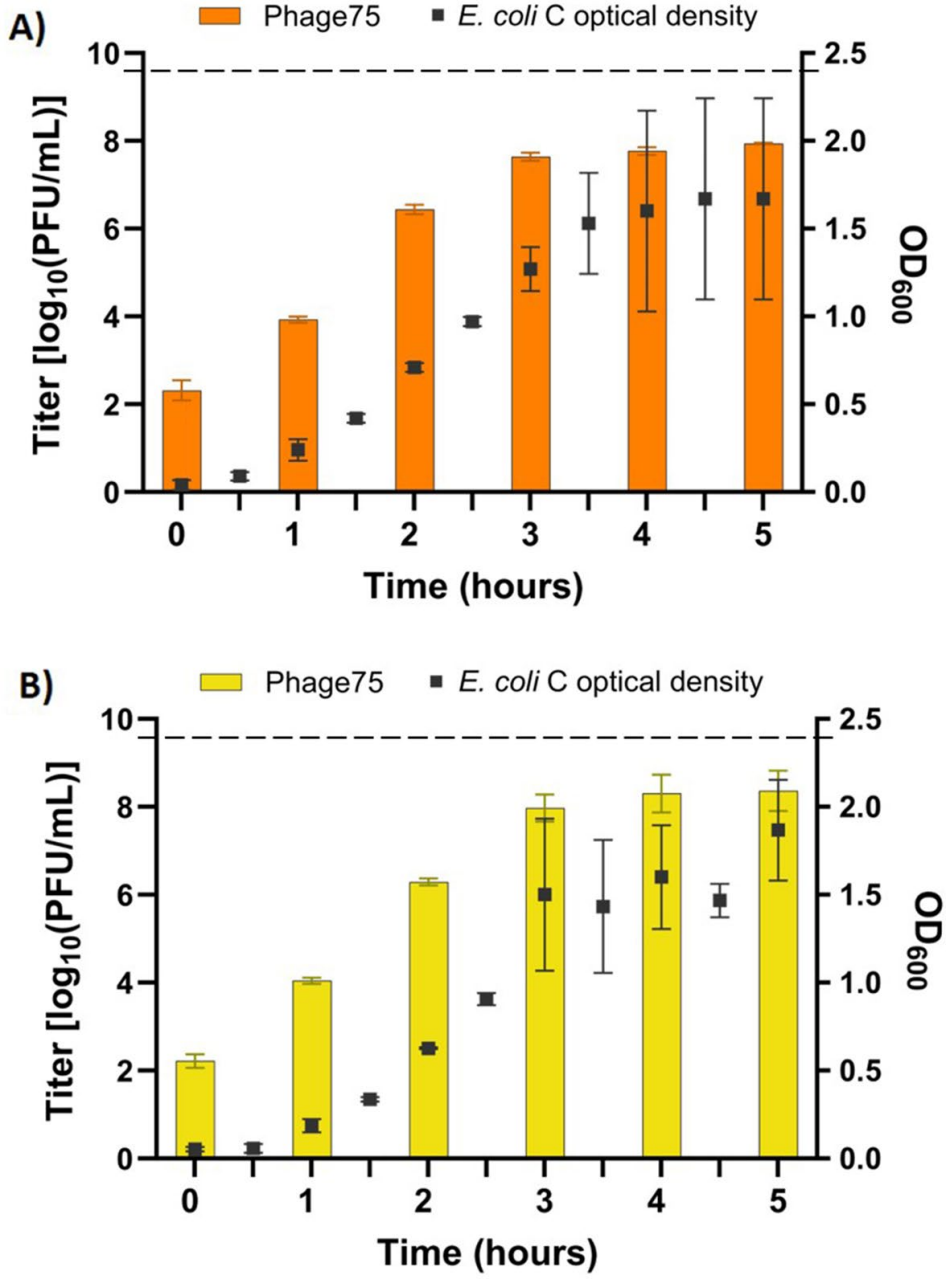
Comparison of Phage75 titer and *E. coli* C optical density (OD). Productions were carried out at 1 L scale in (**A**) LB or, (**B**) APS. A 1 mL sample was removed from production volume every hour and filter sterilized for titer determination. Additionally, an OD_600_ measurement was taken every 30 min. Error bars show standard deviation from the mean (n = 3). Dashed line shows approximate peak Phage75 titer when using ETEC54 host bacteria.

After a successful production in the CellMaker, two alternative bacterial hosts were considered for Phage75 production: *E. coli* B and *E. coli* C with EOP 3.65 × 10^–7^% and 96.50% respectively. Therefore, *E. coli* C was assessed as a surrogate host in 1 L in LB and APS media in shake flasks. However, after 4 h of incubation, achieved titers were 7.77 ± 0.09 and 8.30 ± 0.43 log_10_ PFU/mL in LB and APS respectively, corresponding to a 2.1 and a 1.4 log_10_ PFU/mL reduction compared to the original host, ETEC54 (Fig. 7). Additionally, no population-wide lysis was observed for *E. coli* C.

## Discussion

As bacterial resistance against antibiotics is on-the-rise, phage therapy is attracting more and more attention as the demand for alternative and/or combination therapies against bacterial infections increases. Specifically, strategies and methodologies outlining how to produce high-titer phages efficiently at large-scale will be of upmost importance. The present work establishes a phage-production process for an ETEC-targeting phage, ‘Phage75’, by focusing on high-throughput screening assays and scalable shake flask productions that can reliably translate to bioreactors for industrial scale phage manufacturing.

Based on OD_600_ measurements, assays using 96-well plates can be used to indirectly monitor phage production as host bacteria are lysed^15^. These assays were invaluable to screen complex variables including MOI, media composition (including ion supplementation), temperature, and harvest time to identify optimal production conditions. Specifically, higher phage yields were predicted in nutrient rich media compared to minimal media with shorter harvest times and lower MOI. Anticipated assay conditions resulted in high-titer phage production when translated to shake flasks and bioreactor. These productions at small, medium, and large-scale enabled comparison between variables in more realistic settings.

The effects of media formulations on phage titer were evident across all tested volumes. Interestingly, APS and LB yielded similar phage titers despite having some differences in composition (soy hydrolysate versus peptones and different amounts of yeast extract). This finding is advantageous as APS is not as limited by regulatory requirements as it does not contain animal-based components^43,44^ and has already been used in clinical applications for vaccine and phage preparation^45,46^. Although, these findings may be phage-dependent, alternative protein sources in media have the potential to increase yields and reduce harvest time for phage production^22^. While defined media is recommended for bioprocesses to achieve higher reproducibility and less stringent downstream processing^25^, M9 supplemented with glucose supported lower phage propagation. This is in contrast to another study that showed it rather led to high titer for a different *E. coli*-targeting phage^9^. The reason for this discrepancy is unclear; however, the addition of tryptones to create optimized M9 medium resulted in improved production of Phage75, suggesting a requirement for either more amino acids to be initially present, or even a need for specific amino acids, during its propagation. This is in accordance with previous studies where the addition of tryptophan^47^ and glycine^14^ influenced the titer of *E. coli*- and *S. aureus*-targeting phages respectively. Additionally, phage yields have been improved by substituting glycerol or galactose for glucose^14^, suggesting that different carbon sources may be an important variable to consider.

Screening assays and scalable shake flask experiments suggested monitoring optical density for population-wide lysis can indicate appropriate phage-production endpoints or harvesting. Overall, a similar trend was observed revealing 4 h as a suitable harvest time for Phage75, with 3 to 4 h previously reported for T4^48^. Perhaps unsurprisingly, other studies have indicated that achieving peak titer is medium- and phage-dependent and can occur at different timepoints^22^. This highlights the value of scaled-down investigations as a minimal time investment that can save time in narrowing such conditions before pursuing phage propagation at larger scales. Similar to our findings, these studies show a loss in titer is possible if harvest time is extended to 24 h^22^.

In this study, bacterial re-growth was observed following overnight (22 h) phage propagation (Fig. 4). Though not investigated, potential explanations, aside from genetic mutation that may explain this include: development of phenotypic resistance^49,50^; activation of antiphage defense mechanisms^51^; or the use of quorum sensing to avoid infection^52,53^. However, the proposed production strategy does not require complete eradication of the bacterial population, but rather takes advantage of the balance of allowing host bacteria to survive long enough to divide and create more cells to produce large quantities of phage. As such, regrowth was acknowledged as a potential disadvantage of over-extending phage production including more complicated downstream processing^54^ with no improvement in yields.

To ensure similar outcomes are obtained during scale-up, phage production should be increased by factors of 10 or less^17^, and this was adopted in the present study by using volumes 0.01 L, 0.1 L, and 1 L. Statistical analysis showed Phage75 production is scalable across these three scales and the CellMaker bioreactor, revealing no significant difference in titer (Fig. 6). While some comparisons showed significance, these usually involved the 0.01 L scale, which is not practical for mass phage production and doesn’t negate the scalability otherwise observed between other productions. Overall, shake flasks were useful in determining which conditions continuously led to high titers of Phage75, leading to a robust process capable of translation to higher-volume production, or a bioreactor with different mode of operation. A similar result was achieved for T4 phage when production was scale-up from 0.02 L in shake flasks to 3 L in a bioreactor^16^.

A significant limitation exists as all media types, temperature options, and ion supplements were not examined. Additionally, 96-well plate assays could have been expanded to include phage titrations and infection loads, thereby demonstrating more flexibility of the assay. Another limitation is the use of T4 phage in some aspects of the scale-up process, in the interest of time and consumables, T4 was only used as a benchmark for scale-up and not meant to compare how both phages would behave independently to each condition. A final limitation was the omission of an anti-foaming agent in the CellMaker bioreactor. The extend of phage propagation was likely sacrificed as excessive foaming was observed in the last hour of the bioreactor process. Rapid foaming during phage production usually occurs after population-wide lysis and suggests harvest time^20^. However, phages can also become inactivated due to foaming and bubbling stress^55,56^; negatively impacting yields. A study looking at filamentous phage production suspected reduced yields due to foam^57^. Inevitable foaming during phage production could be mitigated by addition of an antifoaming agent. Antifoam agents may include: fatty acids, sulfonates, silicones, and alcohols^25,56,58^.

A surrogate host (*E. coli* C) was briefly explored for producing Phage75 to avoid growing large quantities of highly pathogenic bacteria (ETEC54) in future productions. *E. coli* C was chosen based on high EOP, but produced significantly less Phage75 than when ETEC54 was host; likely due to insufficient lysis. This raises the question of whether EOP exaggerates future production success since other studies exploring hosts have reported high EOP not translating to high yields^22^. Furthermore, EOP has also been reported to underestimate the lytic activity of phages compared to when in liquid^59^. Therefore, a more suitable approach for selecting a surrogate host could be to carry out additional small-scale productions to confirm or deny any insight gained from EOP experiments. We recommend considering and exploring the use of surrogate hosts at the start of phage production, rather than at the end, to avoid translational issues that may arise throughout the scale-up process.

## Conclusion

Carrying out high-throughput screening assays and shake flask productions are invaluable in outlining scalable and efficient phage production. While these methods are simple, they are practical and provide key insight into which variables and conditions are worth pursuing before initiating larger scales of phage production. This can lead to a robust strategy being determined where comparable phage titers are continuously achieved, even when translated to a bioreactor.

### Supplementary Information

Supplementary Information.

## Data Availability

All data mentioned is included in this published article.
