# Supplementary material for: Investigation into scalable and efficient enterotoxigenic Escherichia coli bacteriophage production
Source: Sci Rep. 2024 Feb 13;14:3618. doi: 10.1038/s41598-024-53276-w (PMC10864315; doi:10.1038/s41598-024-53276-w)
Supplement: Supplementary file 1 — Supplementary Information. [file 41598_2024_53276_MOESM1_ESM.pdf]

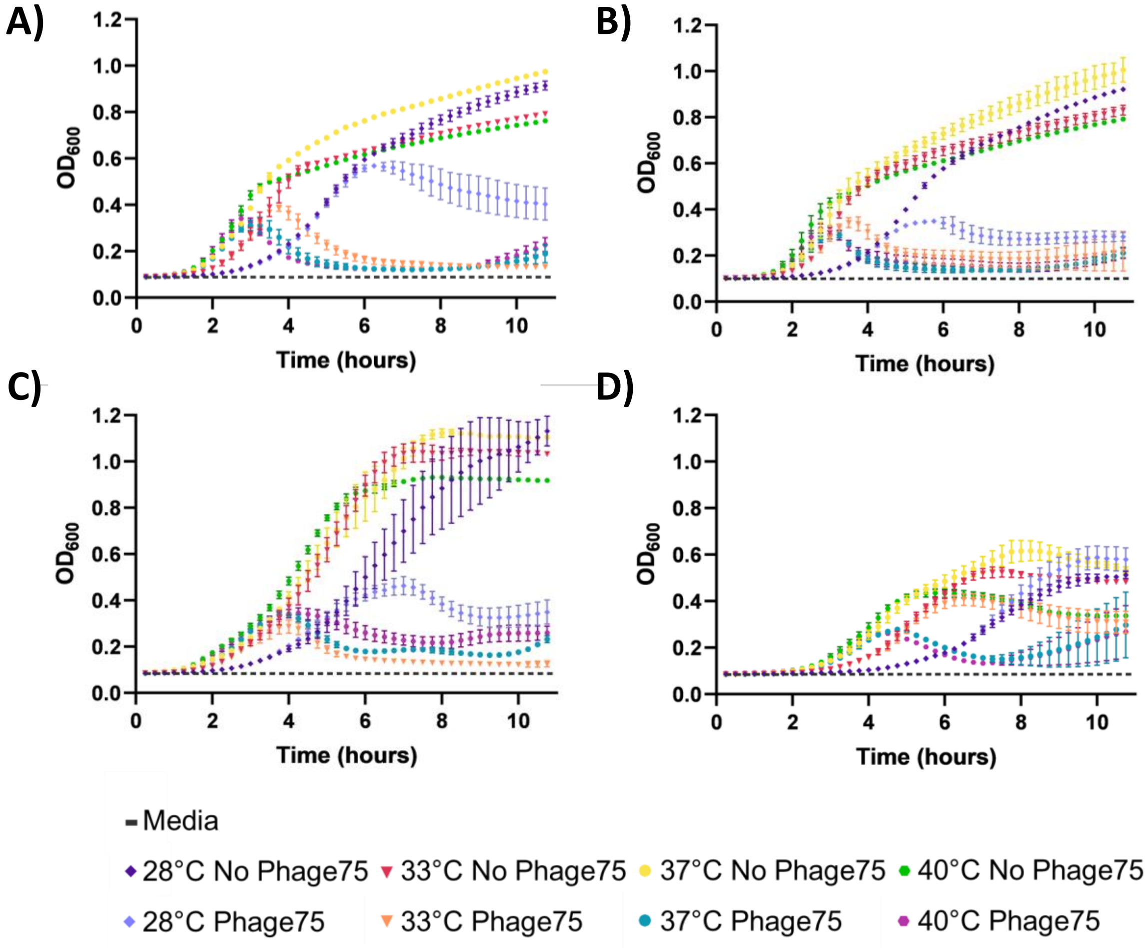

Supplemental Figure S1. Growth curves of ETEC54 in the presence of Phage75, in different media types, at different temperatures. Media type (MOI used): A) LB (10^{-4}); B) APS (10^{-4}); C) Opt. M9 (10^{-3}); D) SM-1 (10^{-3}). Error bars show standard deviation from the mean (n = 3).

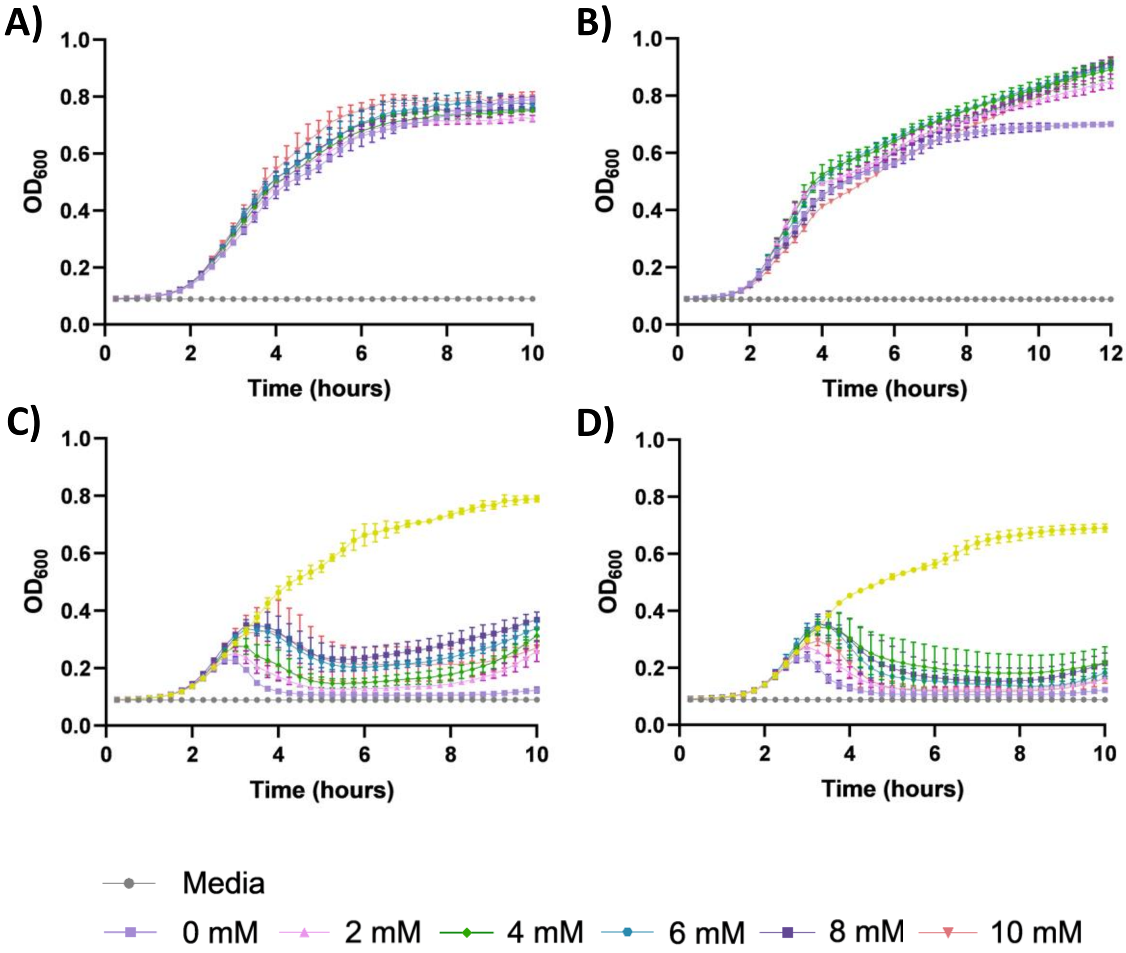

Supplemental Figure S2. Growth curves of ETEC54 in LB supplemented with ions. A) ETEC54 with $CaCl_2$; B) ETEC54 with $MgSO_4$; C) ETEC54 and Phage75 with $CaCl_2$; D) ETEC54 and Phage75 with $MgSO_4$. For graphs C) and D), a 10^{-4} MOI was used with bacterial control (no added phage or ion) being shown in yellow. Error bars show standard deviation from the mean (n = 3).

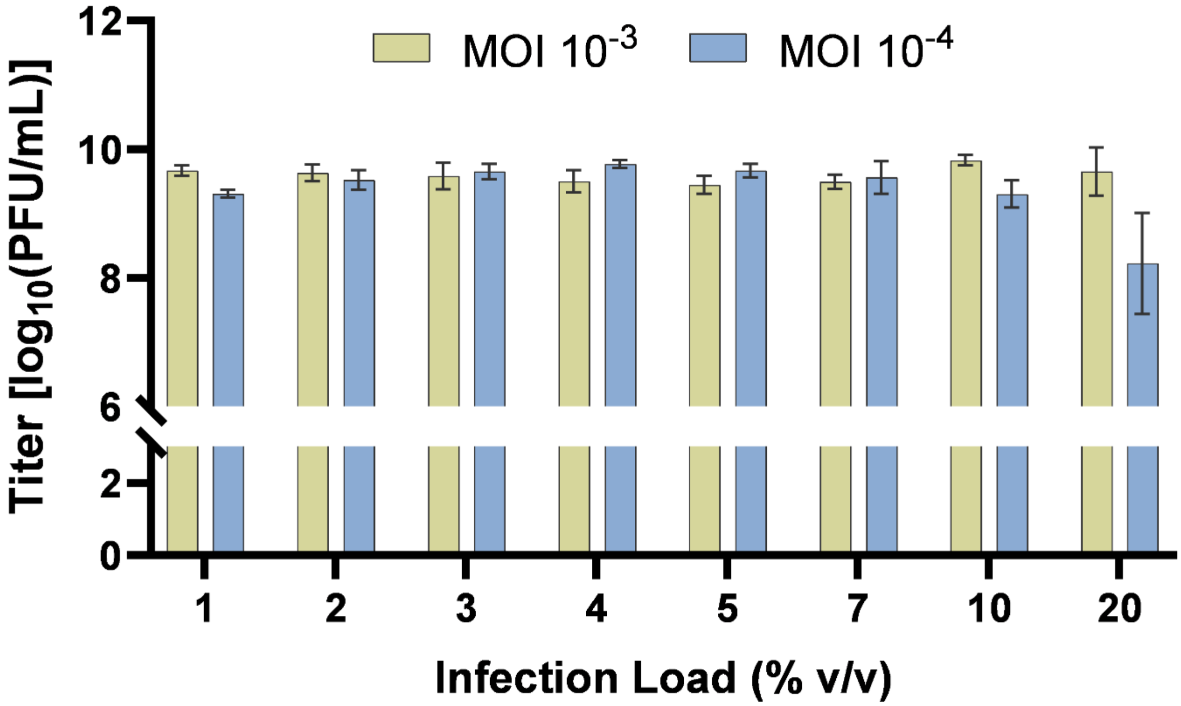

Supplemental Figure S3. Titer comparison between different MOI conditions and infection loads for Phage75 production in ETEC54. Productions were carried out in 0.01 L of LB and harvested after 8 hours. Error bars show standard deviation from the mean (n = 3 to 4).
